# Raphanus sativus (Radish): Their Chemistry and Biology

**DOI:** 10.1100/tsw.2004.131

**Published:** 2004-09-13

**Authors:** Rosa Martha Pérez Gutiérrez, Rosalinda Lule Perez

**Affiliations:** Laboratorio de Investigación de Productos Naturales, Escuela Superior de Ingeniería Química e Industrias extractivas IPN, México D.F., Mexico

**Keywords:** Cruciferae, alkaloids, proteins, polysaccharides, phenolic, sulfur compounds

## Abstract

Leaves and roots of *Raphanus sativus* have been used in various parts of the world to treat cancer and as antimicrobial and antiviral agents. The phytochemistry and pharmacology of this radish is reviewed. The structures of the compounds isolated and identified are listed and aspects of their chemistry and pharmacology are discussed. The compounds are grouped according to structural classes.

### INTRODUCTION

The plant family of Cruciferae contains many important vegetables of economic importance. *Raphanus sativus* L. is originally from Europe and Asia. It grows in temperate climates at altitudes between 190 and 1240 m. It is 30–90 cm high and its roots are thick and of various sizes, forms, and colors (see Fig. 1). They are edible with a pungent taste. Salted radish roots (Takuan), which are consumed in the amount of about 500,000 tons/year in Japan, are essentially one of the traditional Japanese foods. The salted radish roots have a characteristic yellow color, which generates during storage.

This specie is used popularly to treat liver and respiratory illnesses[1]. The antibiotic activity of its extracts and its time persistence validates its effectiveness in microbial sickness as reported in traditional medicine. The root's juice showed antimicrobial activity against *Bacillus subtilis, Pseudomonas aeruginosa*, and *Salmonella thyphosa*. The ethanolic and aqueous extracts showed activity against *Streptococcus mutans* and *Candida albicans*. Aqueous extract of the whole plant presents activity against *Sarcinia lutea* and *Staphylococcus epidermidis*[2]. Aqueous extract of the leaves showed antiviral effect against influenza virus. Aqueous extract of the roots showed antimutagenic activity against *Salmonella typhimurium* TA98 and TA100. In this review, the metabolites produced by *R. sativus* are presented according to structural classes. (See also Tables 1 through 10 at the end of this paper.)

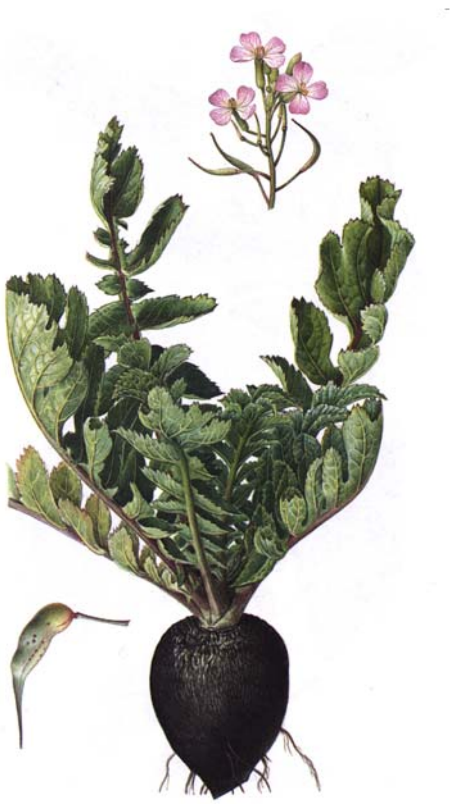

**FIGURE 1**

## CHEMICAL CONSTITUENTS

### Alkaloids and Nitrogen Compounds

Alkaloid and nitrogen compounds present in the roots were pyrrolidine, phenethylamine, N-methylphenethylamine, 1,2´-pyrrolidin-tion-3-il-3-acid-carboxilic-1,2,3,4-tetrahydro-β-carboline, and sinapine[3,4,5]. Cytokinin (6-benzylamino-9-glucosylpurine) is a major metabolite of 6-benzylaminopurine (6-BAP) in the root radish. A minor metabolite of 6-BAP from radish has been identified as 6-benzylamino-3-β-D-glucopyranosylpurine[6]. Total amino acids were 0.5% of dry wt; with proline (0.5%) as the major constituent, methionine and cystine were present in traces (0.02%). Diamines as diaminotoluene (2,4-D), 4,4´-methylenedianiline (4,4-D), and 1,6-hexanediamine (1,6-D) were isolated in the period of germination of young radish seeds. Production of thiamine is higher during germination radishes[7].

Total protein was 6.5%[8]. Two chitinases, designated RRC-A and RRC-B, were isolated from radish roots. Both compounds had a molecular weight of 25 kDa[9]. N-Bromosuccinimide and di-Et-pyrocarbonate inhibited the activities of both chitinases.

Arabinogalactan proteins (AGPs) were isolated from primary and mature roots of the radish. These were composed mainly of L-arabinose and D-galactose. Structures of the carbohydrate moieties of the root were essentially similar to those isolated from seeds and mature leaves in that they consisted of consecutive (1→3)-linked β-D-galactosyl backbone chains having side chains (1→6)-linked β-D-galactosyl residues, to which α-L-arabinofuranosyl residues were attached in the outer regions. One prominent feature of the primary root AGPs was that they contained appreciable amounts of L-fucose[10].

Two L-arabino-D-galactan–contained glycoproteins were isolated from the saline extract of mature radish leaves; both contained L-arabinose, D-galactose, L-fucose-4-O-methyl-D-glucuronic acid, and D-glucuronic acid residues. Degradation of the glycoconjugates showed that a large proportion of the polysaccharide chains is conjugated with the polypeptide backbone through a 3-O-D-galactosylserine linkage[11].

Arabino-3,6-galactan associated with a hydroxyproline-rich protein portion and carried a unique sugar residue, α-L-fucopyranosyl-(1-2)-α-L-arabinofuranosyl[12].

Stigma glycoproteins heritable with S-alleles (S-glycoproteins) were detected in *R. sativus*. Two main glycoproteins appeared on the SDS-gel electrophoretic pattern. Their molecular weights were established to be 15,000 and 100,000 Da. The carbohydrate fraction of the glycoprotein consisted of arabinose 17.3%, galactose 19.1%, xylose 8.1%, mannose 5.4%, glucose 23.7%, and rhamnose or fucose 26.4%. In the stigma surface diffusate of *R. sativus*, the content of protein was established to be 16% and that of carbohydrate was 11%[13].

The *R. sativus acanthiformis* showed two ferredoxin isoproteins indicating that plants have multiple genes for ferredoxin. The relative abundance of the isoproteins varied with leaf stage[14]. In the isoprotein isolated from roots of the radish, the amino acid composition and N-terminal sequence were different from those of radish leaf ferredoxin.

Polypeptides RCA1, RCA2, and RCA3 were purified from seeds of *R. sativus*. Deduced amino acid sequences of RCA1, RCA2, and RCA3 have agreement with average molecular masses from electrospray mass spectrometry of 4537, 4543, and 4532 kDa, respectively. The only sites for serine phosphorylation are near or at the C terminal and hence adjacent to the sites of proteolytic precursor cleavage[15].

Cysteine-rich peptides (Rs-AFP1 and Rs-AFP2) isolated from *R. sativus* showed peptides 6, 7, 8, and 9 comprising the region from cysteine 27 to cysteine 47[16]. Protein AFP1 isolated from radish showed peptide fragments (6-mer, 9-mer, 12-mer, and 15-mer)[17].

Proteins RAP-1 and RAP-2 were isolated from Korean radish seeds. The molecular mass of the two purified was established to be 6.1 kDa (RAP-1) and 6.2 kDa (RAP-2) by SDS-PAGE and 5.8 kDa (RAP-1) and 6.2kDa (RP-2) by gel filtration chromatography[17].

### Coumarins

Hydroxycoumarins aesculetin and scopoletin were also identified[18].

### Enzymes

A number of enzymes are present in both the cytoplasm and the cell wall, and in some cases it has been shown that the cell wall isozymes differ from those of the cytoplasmic[19]. When radish seedlings are grown in the dark, β-fructosidase (βF) first accumulates in the cytoplasm, then slowly increases in the cell wall. Charge heterogeneity of cytoplasmic enzymes resides in the polypeptides, while the formation of the basic cell wall occurs as a result of post-translational modifications that can be inhibited by tunicamycin[20].

Cysteine synthase (EC 4.2.99.8) was purified to near homogeneity (275-fold) in 11.5% yield from mature roots. It was relatively stable, retaining most of its activity in standing for several days at room temperature[21].

A basic β-galactosidase (β-Galase) has been purified from imbibed radish. This enzyme, consisting of a single polypeptide with an apparent molecular mass of 45 kDa and pI values of 8.6 to 8.8, was maximally active at pH 4.0 on p-nitrophenyl β-D-galactoside and β-1,3-linked galactobiose. Radish seed and leaf arabino-3,6-galactan-proteins were resistant to the β-Galase[22]. β-Amylase[23], together with peroxidase c or paraperoxidase[24], which is an isoenzyme, were also isolated from Japanese radish roots.

A hydroxycinnamoyltransferase (EC 2.3.1.-), which catalyzes *in vivo* the formation of 1,2-di-O-sinapoyl-β-D-glucose, was isolated from the radish. Cotyledons exhibited activities of 1-O-acyl-glucose–dependent acyltransferases, 1-sinapoyl-glucose:L-malate sinapoyltransferase (SMT), and 1-(hydroxycinnamoyl)-glucose:1-(hydroxycinnamoyl)glucose-hydroxyl cinnamoyl-transferase (CGT), showing contrary developments depending on light conditions. Light-grown seedlings showed high L-malate sinapoyltransferase and low 1-(hydroxycinnamoyl)glucose-hydroxyl cinnamoyl-transferase activities, while dark-grown seedlings showed low L-malate sinapoyltransferase and high 1-(hydroxycinnamoyl)glucose-hydroxyl cinnamoyl-transferase activities[25].

Catalase and glutathione reductase activities increased considerably in the root and leaves after 24-h exposure to cadmium, indicating a direct correlation with Cd accumulation. PAGE enzyme activity staining revealed several superoxide dismutase isoenzymes in leaves. The main response may be via activation of ascorbate-glutathione cycle for removal of hydrogen peroxide or to ensure availability of glutathione for synthesis of Cd-binding proteins[26].

A γ-glutamyl transpeptidase was found. It catalyzed the release of CySH-Gly from glutathione, the release of alanine from γ-glu-Ala, and the formation of γ-glutamyl dipeptides. A dipeptide formed from S-methylcysteine and glutathione or γ-glu-Ala was characterized as γ-glutamyl-S-methylcysteine[27].

Two cationic isoperoxidases (C1 and C3) and four anionic isoperoxidases (A1, A2, A3n, and A3) were isolated from Korean *R. sativus* L. root. All the six isoperoxidases are glycoproteins composed of a single polypeptide chain. The molecular weights of C1, C3, A1, and A2 were ca. 44,000, while anionic isoperoxidase A3n and A3 have molecular weights of 31,000 and 50,000, respectively. N-terminal amino acid sequences were determined for A1, A3n, and C3, while A2 was found to have a blocked terminal residue[28]. Analysis of digested products of the two major N-glycans of C3 suggested that core-fucosylated trimannosylchitobiose may contain a different linkage from the typical α-1,6 of native N-linked oligosaccharide[29].

Thiamin-binding substances were found in the radish. There were two kinds of compounds; one was heat labile and Pronase sensitive, and the other was heat stable and Pronase resistant. It would be inferred that the former is protein and the latter is a nonprotein compound[30].

βF is an isozyme (glycoprotein) found in the cytoplasm and cell walls of the radish. The nonglycosylated cytoplasmic and cell wall βF forms have the same relative molecular mass, but glycosylated forms have different oligosaccharide side chains with respect to size and susceptibility to α-mannosidase and endoglycosidase D digestion[31].

7-Glucoside de zeatin, isolated from radish cotyledons, occurs naturally as glycoside with β-glucose as substituent. A large number of derivatives of purine are glucosylated, but adenine derivatives with alkyl side chains at least three carbon atoms in length at position N6 are preferentially glucosylated[20].

### Gibberellins

The bolting (stem elongation accompanying flowering) of *R. sativus* L. cv. Taibyo-sobutori requires cold treatment (Vernalization) and subsequent long-day conditions. It has been suggested that gibberellins (GAs) might be involved in the control of bolting. Eleven gibberellins were identified in extracts of mature seed as 13 hydroxy-GAs [$GA_1$, 3-*epi*-$GA_1$, $GA_8$, $GA_{17}$, $GA_{19}$, $GA_{20}$, and a new GA, 12α-hydroxy-$GA_{20}$ ($GA_{77}$)] and four non-13-hydroxy-GAs [$GA_9$, $GA_{24}$, 12β-hydroxy-$GA_{24}$, $GA_{25}$]. The major GAs were $GA_8$, $GA_{20}$, and $GA_{77}$[32].

### Glucosinolates

Glucosinolates are very stable water-soluble precursors of isothiocyanates. The relatively nonreactive glucosinolates are converted to isothiocyanates on wounding of the radish. The tissue damage releases myrosinase (EC 3.2.3.1), a glycoprotein that is physically segregated from its glucosinolate substrates. Large variations in myrosinase-specific activity have been reported in various Cruciferous plant sources. Myrosinase, purified to homogeneity from daikon, has a specific activity of 280 μMol/min/mg protein with sinigrin as a substrate[33]. Glucosinolate contents of seed of radish cultivar ranged from 37–87 μmol/g seed. The 5-vinyl-2-oxazolidinethione, 3-butenyl, 4-pentenyl, and phenethyl isothiocyanate were found in industrially extracted rapeseed oils. The compounds were hydrolysis products from glucosinolates present in the seed[34].

Desulfoglucosinolates are formed by enzyme desulfation of endogenous glucosilates. The indole glucosinolates, 4-methoxy-3-indolylmethyl glucosinolate and 1-methoxy-3-indolylmethoxy glucosinolate, were absent in seed whereas 4-hydroxy-3-indolymethyl glucosinolate was found in highest concentration in the seeds. The 3-indolymethyl glucosinolate was found in low levels in seed, but was the dominant indole glucosinolate in the leaf[35].

### Oil Seed Components

The seeds of the radish contain a high percentage of oil. Chromatographic analysis of these oils showed clearly their complete similarities to cottonseed oil[36]. The steam volatile constituents of fresh radish of Japanese and Kenyan origin have been studied. The overall pattern of compounds in the two materials was similar. Major components are pentyl hexyl, 4-methylpentyl isothiocyanate, dimethyl disulfide, methyl methanethiolsulfinate, and 1-methylthio-3-pentanone[37]. Oil radish seeds contained 1.21 μmol of total alkenylglucosinolates (AG/g), consisting mostly of progoitrin and gluconapin[38].

### Organic Acids

Four major organic acids are present in the roots of the radish: oxalic, malic, malonic, and erythorbic acid. Lipid total was 1.23%[8]. Major fatty acids in seed lipids were erucic, oleic, linoleic, and linolenic acids. Major fatty acids in radish family lipids were linolenic acid (52–55%), followed by erucic acid (30–33%), and palmitic acid (20–22%)[39]. Also identified were stearic acid from petroleum ether extracted from powdered *R. sativus* seeds. Glutamic acid is found in pickled daikon (20–100 mg%)[40].

### Phenolic Compounds

The content of phenolic acids in the roots of the radish were much smaller than in the leaves. Radishes and horseradish showed caffeic, *p*-coumaric, ferulic, hydroxycinnamic, *p*-hydroxybenzoic, vanillic, salicylic, and gentisic acid[18]. Sinapic acid esters (1-sinapolyglucose, sinapoyl-L-malate, and 6,3´-disinapoylsucrose), kaempferol glycosides, and free malic acid were isolated from cotyledons of *R. sativus* seedlings[41].

Among the anthocyanins, pelargonidine and cyanidine were responsible for red and violet color in corollas and roots in all inbred progenies. The absence of pelargonidine and cyanidine resulted in a white color. The flavonoid, quercetine, was also found in both corolla and root[42]. Anthocyanins extracted from epidermal tissue resulted in juices with fairly low initial ˚Brix (1.3˚), containing 400 mg anthocyanin/100 ml. This compound provided color similar to FD&C Red#40. Radish concentrate extract represents a promising natural alternative to the use of FD&C Red#40[43].

Other purple root pigment isolated from progeny radish was an ester of cyaniding triglucoside and three kinds of cinnamic acids. The triglucoside was identified as the 2-diglucoside-5-monoglucoside of cyaniding (Rubrobrassicin)[44]. Other anthocyanins obtained from red radish are two diacylated pelargonidin 3-O-[2-O-β-glucopyranosyl)-6-O-(*trans*-p-coumaroyl)-β-glucopyranoside]-5-O-(6-O-malonyl-β-glucopyranoside) and pelargonidin 3-O-[2-O-(β-glucopyranosyl)-6-O-(*trans*-p-feruloyl)-β-glucopyranoside]-5-O-(6-O-

malonyl-β-glucopyranoside) and other monoacylated anthocyanins pelargonidin 3-O-[2-O-β-glucopyranosyl)-6-O-(*trans*-p-coumaroyl)-β-D-glucopyranoside]-5-O-(β-glucopyranoside) and pelargonidin 3-O-[2-O-β-glucopyranosidel)-6-O-(*trans*-p-feruloyl)-β-glucopyranoside]-5-O-(β-glucopyranoside). Pelargonidin-3-diglucosido-5-monoglucoside is known as raphanusin[45].

The major anthocyanins of radishes are pelargonidin-3-sophoroside-5-glucoside acetylated with malonic acid and either ferulic or p-coumaric acid. Cinnamic acid acylation site for radish anthocyanins was determined to be at position 6 of glucose-1 of the sophorose substituents by one- and two-dimensional $^{1}$HNMR-$^{13}$CNMR[46]. Also 7-glucoside-pelargonidin has been identified in *R. sativus*[47]. This compound was stable at 60˚C and under light, may be used as a food colorant[48].

Kaempferol-7-O-rhamnoside, isorhamnetin-7-O-rhamnoside, quercetin-7-O-rhamnoside, kaempferol-3-glucoside-7 rhamnoside, kaempferol-7-glucoside-3 rhamnoside, quercetin-7-O-arabinoside-3-glucoside, and quercetin-7-glucoside-3 rhamnoside were isolated from *R. raphanistrum*[49]. Radishes have a high content of flavonoids as quercetin, kaempferol, myricetin, apigenin, and luteolin[50]. Malvidin-3,5-diglucoside was produced from the callus of radish via tissue cultivation. The callus contains 16.4% (dry wt) pigments[51].

### Pigments

Salted radish roots have a characteristic yellow color, which generates during storage. 4-Methylthio-3-butenyl-glucosinolate (4-MTBG) is the substrate of the main pungent principle of radish and is one of the essential factors for the formation of the yellow pigment. The yellow compound 1-(2´-pyrrolidinethion-3´-yl)-1,2,3,4-tetrahydro-β-carboline-3-carboxilic acid is presumed to have been the condensation product from the degradation of 4-methylthio-3-butenylisothiocyanate and L-tryptophan, which carboline compound is considered to play an important role in the formation of the yellow pigment in salted radish roots[52].

### Polysaccharides

Pectic substances were extracted from the leaves with oxalate buffer of pH 4.25 as weakly acidic pectic polysaccharide (WAP) and pectic acid. WAP was appreciably hydrolyzed by exo- and endopolygalacturonases and the galacturonic acid content (17.3–25.8%) was much lower than the pectic acids, though the neutral sugar components of both pectic substances were almost the same. The arabinose-galactose side chains were very long or highly branched in pectine compared with those in pectic acids. These compounds are probably inherent pectic components of the cell walls of the vegetables[53]. Rhamnose, glucose, and xylose were also isolated. Lipopolysaccharides (LPS) were isolated from radish roots[54].

### Proteoglycan

An L-arabino-D-galactan–contained proteoglycan was isolated from hot phosphate-buffered saline extract of radish seeds by ethanol fractionation. The proteoglycan consisted of 86% of a polysaccharide component–contained L-arabinose and D-galactose as major sugar constituents, together with small proportions of D-xylose, D-glucose, and uronic acids, and 9% of a hydroxyproline-contained protein. Arabinogalactan from radish seed had a high content (81%) of L-arabinose and its basic structure seemed to be similar to that of the polysaccharide component of the proteoglycan[55].

### Sulfur Compounds

Radish leaves contain only one of the sulfonium diateroisomers of S-adenosylmethionine (AdoMet), which has a remarkable variety of biochemical functions. It is an allosteric enzyme effector and a precursor of spermine biosynthesis, spermidine, and ethylene. It is also the methyl group donor for most biological transmethylation reactions, wherein transfer of its methyl group converts AdoMet to the homocysteine analog (AdoHcy). Much of the chemistry and biochemistry of AdoMet derives from the fact that it is a sulfonium compound[56]. 1-(2´-Pyrrolidinethion-3´-yl)-1,2,3,4-tetrahydro-β-carboline-3-carboxilic acid was found in radish root. This carboline compound is considered to play an important role in the formation of the yellow pigment in salted radish roots.

### Other Constituents

β-Carotene was isolated from radish. Vitamin C content in fresh hotbed radishes ranged from 17.95–27.86 mg%[57]. Also identified was β-sitosterol from *R. sativus* seeds[40]. The contents of raphanusol A and B in radish increased at the lighted side and decreased in the shaded side. The differential distribution of raphanusol A and B in the hypocotyls is closed correlated with growth suppression at lighted side[58].

## BIOLOGICAL ACTIVITIES

### Allergic Contact

In the radish, the allyl isothiocyanate released enzymically from simigrin, a thioglycoside, was identified as a possible sensitizing substance. In some cases, it can produce allergic contact and dermatitis[59]. The leaves of this plant also contained glucoparin that produced allergic contact.

### Antimicrobial Activity

Crude juice of the radish inhibited the growth of *Escherichia coli, Pseudomonas pyocyaneus, Salmonella typhi,* and *Bacillus subtilis in vitro*. This common plant may be an important source of antimicrobial substances[60]. The cysteine-rich peptides (Rs-AFP1 and Rs-AFP2) isolated from *R. sativus* showed substantial antifungal activity against several fungal species with minimal inhibitory concentration (MIC) of 30–60 µg/ml. Both Rs-AFPs are among the most potent antifungal proteins characterized. Moreover, their antibiotic activity shows a high degree of specificity to filamentous fungi[16]. The active region of the antifungal protein appears to involve β-strands 2 and 3 in combination with the loop connecting those strands[61]. Rs-AFP1 and Rs-AFP2 are highly basic oligomeric proteins composed of small (5-kDa) polypeptides that are rich in cysteine. These proteins are located in the cell wall and occur predominantly in the outer cell layers lining different seed organs. Moreover, Rs-AFPs are preferentially released during seed germination after disruption of the seed coat[62]. Two purified antifungal proteins RAP-1 and RAP-2 isolated from Korean radish seeds (*R. sativus*) exhibited growth-inhibitory activities against *Candida albicans* and *Saccharomyces cerevisiae*[63]. The protein AFP1 isolated from the radish showed antifungal activity against *Fusarium culmorum*[17].

Caffeic acid showed antifungal properties *in vitro* against *Helminthosporium maydis.* It has antibacterial, antifungical activities. Ferulic acid is active against *Sytaphylococcus aureus, Bacillus subtilis, Corynebacterium, diphtheria, Aspergillus niger*, and *Candida albicans*. These acids displayed antibacterial activity against Gram-positive bacteria *Bacillus subtilis* and *Staphylococcus aureus*, and the Gram-negative *Escherichia coli* and *Kliebsiella pneumoniae*. The MIC values were 1.56–3.13 µg/ml.

These *p*-hydroxybenzoic acid (hydroxycinnamic, *p*-hydroxybenzoic) showed marked activity against Gram-positive bacteria.

The inoculation of sliced daikon roots with the bacterium *Pseudomonas cichorii* induced the formation of several antifungal compounds including brassinin, methoxybrassinin, spirobrassinin, and 3-indolecarbaldehydes[64].

The radish released biocidal compounds, mainly isothiocyanates, produced during the enzymic degradation of glucosinolates present in the plant cell. The highest fungicidal activity depended on concentration of isothiocyanates[65].

### Antioxidative Activity

The red radish pigment (pelargodinin-3-sophoroside-5-glucoside) had almost the same antioxidative activity as BHT at the same concentration. The inhibition ratio could reach more than 93% by the 0.01% pigment addition[66]. Also, the caffeic acid showed antioxidative activity.

### Antitumor Activity

A neutral fraction of kaiware radish extract aqueous *in vitro* showed proliferation inhibition of mouse embryo fribroblast 3T3 cells and papovavirus SV40 transformed 3T3 cells with $IC_{50}$ of 17.4 and 8.7 µg/ml[67]. Diaminotoluene (2,4-D) showed highest cytotoxic activity against He-La cells, 4,4´-methylenedianiline (4,4-D) intermediate, and 1,6-hexanediamine (1,6-D) lowest cytotoxicity. However, the phytotoxicity decreased in order of 4,4-D >2,4-D>1,6-D[7].

### Antiviral Activity

Caffeic acid and pelargonidin are virucidal for several enveloped viruses[41]. The lipopolysaccharides showed antiherpes activity.

### Calmodulin Antagonists

The polypeptides RCA1, RCA2, and RCA3 inhibit chicken gizzard calmodulin-dependent myosin light kinase assayed with a myosin-light chain-based synthetic peptide substrate[15].

### Growth Inhibitors

The hypocotyls *cis*- and *trans*-raphanusanins and 6-methoxy-2,3,4,5-tetrahydro-1,3-oxazepin-2-one (raphanusamide) were isolated from radish. *Cis*- and *trans*-raphanusanins inhibited the hypocotyls growth at concentrations higher than 1.5 µM and raphanusamide at concentrations higher than 20 µM[68]. Growth-inhibitor 2-thioxothiazolidine-4-carboxilic acid was isolated from acetone extract of light-exposed seedlings of Sakurajima radish. It inhibited the growth of hypocotyl sections of etiolated Sakurajima radish and intact hypocotyls of etiolated lettuce seedlings at concentrations greater than 3 mg/ml. Gibberellins were identified in extracts of mature seed radish and might be involved in the control of bolting (stem elongation accompanying flowering) of *R. sativus*[32].

### Hypotensive

Sinapine was extracted with methanol. It is a hypotensive constituent of laifuzi (*Semen raphani*) and seed of *R. sativus*[5].

### Platelet Aggregation Inhibitor

The 6-methyl-sulfinylhexyl-isothiocyanate (MS-ITC) was isolated from wasabi horseradish (Japanese domestic) as a potential inhibitor of human platelet aggregation *in vitro*. It is a potential inducer of GST (glutathione S-transferase). In the mechanism of MS-ITC, the isothiocyanate moiety of MS-ITC plays an important role for antiplatelet and anticancer activities because of its high reactivity with sulfhydryl (-SH) groups in biomols (GSH, cysteine, residue in a certain protein)[69].

### Immunological Properties

The AGPs isolated from the radish showed immunological properties. Radish AGPs R-I, R-II, crude fraction R-C, and turnip AGP B-II reacted with eel anti-H serum, indicating that these AGPs shared common antigenic determinants[70]. The root's AGPs were composed mainly of L-arabinose and D-galactose, but were distinguishable from each other in their contents of L-fucose as well as of protein and hydroxyproline. Structures of AGPs from the root, seeds, and mature leaves were essentially similar[71]. Proteoglycan from radish leaves and seeds appeared to share common antigenic determinant[55].

### Phytoalexins

The inoculation of sliced daikon roots with the bacterium *Pseudomonas cichorii* induced the formation of several antifungal compounds including brassinin, methoxybrassinin, spirobrassinin, and 3-indolecarbaldehydes[64].

### Pungent Principle

The pungent principle extracted from the radish root is *trans*-4-methylthio-3-butenyl-isothiocyanate. Also isolated was the *cis*-isomeride, in a *trans–cis* ratio of 4:1[72]. 2-Thioxo-3-pyrrolidinecarbaldehyde (TPC) is a major product generated from the pungent principle of radish. This compound possesses antimicrobial activity with the MIC against fungi and bacteria ranging from 50–400 µg/ml, while yeasts were more resistant. The antifungal and antibacterial actions were due to the sporicidal and bactericidal activities. A dose-dependent inhibition of the uptakes of both oxygen and the precursors for RNA and DNA was observed, suggesting that TPC caused damage to the mitochondrial functions and biosynthetic systems[73].

### Serological Activity

AGPs were presumably responsible for expression of the serological activity. In their immunological reactions with rabbit antiradish leaf AGP antibody, the root AGPs were shown to share common antigen determinant with those of seed and leaf AGPs[10]. Arabino-3,6-galactan associated with a hydroxyproline-rich protein portion, which might be responsible for the serological H-like activity of the

AGPs[12]. Two L-arabino-D-galactan–contained glycoproteins having potent inhibitory activity against eel anti-H agglutinin were isolated from the saline extract of mature radish leaves[70].

### Intestine Motility Stimulation

The effect of radish aqueous extract at doses of 10 µg/ml to 2 mg/ml caused a dose-dependent increase in contractions of the duodenum, jejunum, and ileum. Ileal contraction was remarkably inhibited by pretreatment of atropine ($10^{-7}$ M) by 10 min. Oral administration of radish extract (300–500 mg/kg body wt) to mice improved the intestinal transit of charcoal and this was significantly attenuated by co-administration of atropine (50 mg/kg). These results suggest that radish extract stimulates gastrointestinal motility through activation of muscarinic pathways[74]. Scopoletin is an antispasmodic agent.

### Cardiovascular Disease Prevention

Radish powder decreased the lipid levels by increasing the fecal excretion of total lipids, triglycerides, and total cholesterol. Catalase and glutathione peroxidase (GSH-Px) activities in red blood cell (RBC) were most remarkably increased by radish. Superoxide dismutase (SOD), catalase, and GSH-Px activities in the liver were increased by radish powder. Xanthine oxidase (XOD) activities in the liver were decreased by radish. Flavonoids and vitamin C in radish may inhibit lipid peroxidation, promote liver and RBC catalase, and inhibit XOD activities in animals tissues. Radish can be recommended for the treatment and prevention of diseases such as cardiovascular disease and cancer and for delaying aging[75].

### Other Activities

Lipopolysaccharides (LPS) were isolated from radish having a macrophage activating with $ED_{50}$ of 0.4–100 ng/ml. These compounds can be used as antidiabetic agents in pharmaceutical or veterinary fields. Also the LPS showed analgesic activity[54].

**TABLE 1**
**Alkaloids and Nitrogen Compounds Isolated from Radish**

| Structure | Source | Activities |
|---|---|---|
|  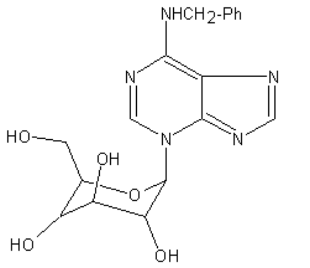 (6-Benzylamino-9-glucosylpurine) | Cotyledon[6] | Cytokinin activity[6] |
|  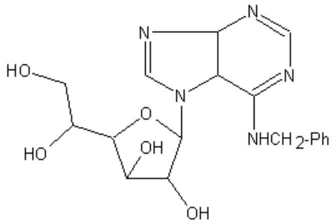 6-Benzylaminopurine | Cotyledon[6] | Cytokinin activity[6] |
| 2,4-Diaminotoluene<br>4,4´-Methylenedianiline<br>1,6-Hexanediamine | Germination of young radish seeds[7] | Cytotoxic activity against He-La cells[7] |
|  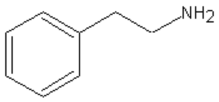 Phenethylamine | Leaves[3] | Skin irritant and possible sensitizer[5] |
|  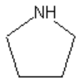 Pyrrolidine | Radish leaves[4] | |
|  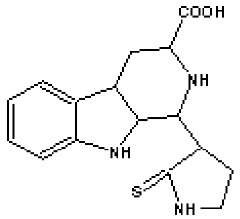 1-(2´-Pyrrolidinethion-3´-yl)- 1,2,3,4-tetrahydro-β-carboline-3-carboxilic | Leaves[3] | |
|  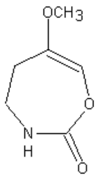 Raphanusamide | All tissue[68] | Inhibited growth of etiolated radish[68] |

**TABLE 1 (CONTINUED)**
**Alkaloids and Nitrogen Compounds Isolated from Radish**

| Structure | Source | Activities |
|---|---|---|
|  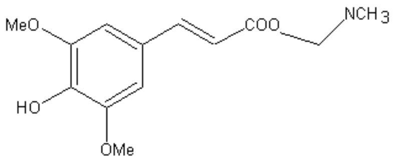 Sinapine | Seeds[5] | Hypotensive[76] |
|  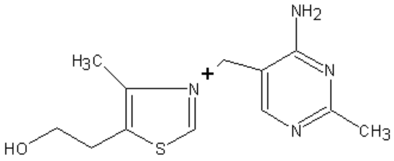 Thiamine | Germination[30] | Essential vitamin[30] |

**TABLE 2**
**Coumarins Isolated from Radish**

| Structure | Source | Activities |
|---|---|---|
|  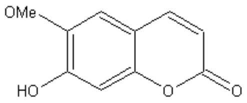 Aesculetin | Root[18] | Antifungical[76] |
|  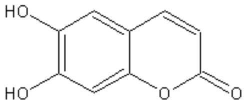 Scopoletin | Root[18] | Antispasmodic[76] |

**TABLE 3**
**Enzymes Isolated from Radish**

| Structure | Source | Activities |
|---|---|---|
| Anionic isoperoxidases: A1, A2, A3n, and A3 | Leaves[29] | |
| β-Amylase | Roots[23] | |
| Arabinogalactan proteins | Leaves, primary and mature roots[10] | Serological activity[10] |
| Catalase | Leaves[26] | |
| Cationic isoperoxidases: C1 and C3<br>Anionic isoperoxidases: A1, A2, A3n, and A3 | Korean radish roots[28]) | |
| Cysteine synthase | Mature roots[21] | Antifungical[21] |
| β- Galactosidase | Seeds[22] | |
| 7-Glucoside de zeatin | Cotyledons[20] | Cytokinin activity[20] |
| γ-Glutamyl transpeptidase | Bulb[27] | |
| Glutathione reductase | Roots and leaves[27] | Antioxidant activity; lowering lipid levels[26] |
| Hydroxycinnamoyltransferase | Cotyledons of radish[25] | |
| β-Fructosidase (βF) | Mature leaves[31] | |
| Peroxidase c | Japanese radish roots[24] | |
| Superoxide dismutase | Roots and leaves[75]) | Antioxidant activity; lowering lipid levels |
| 1-(Hydroxycinnamoyl)glucose-hydroxyl cinnamoyl-transferase (CGT) | Cotyledons[25] | Effect on light-grown seedling[25] |
| L-Malate sinapoyltransferase (SMT) | Cotyledons[25] | Showed effect on light-grown seedling[25] |

**TABLE 4**
**Gibberellins Isolated from Radish**

| Structure | Source | Activities |
|---|---|---|
|  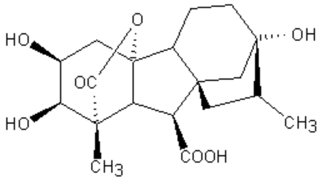 GA$_8$ | Mature seed of radish[32] | Control of bolting[32] |
|  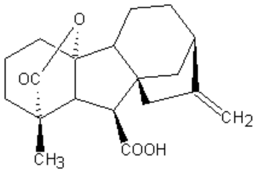 GA$_9$ | Mature seed of radish[32] | Control of bolting[32] |

**TABLE 4 (CONTINUED)**
**Gibberellins Isolated from Radish**

| Structure | Source | Activities |
|---|---|---|
|  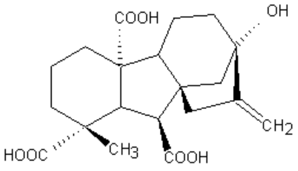 $GA_{17}$ | Mature seed of radish[32] | Control of bolting[32] |
|  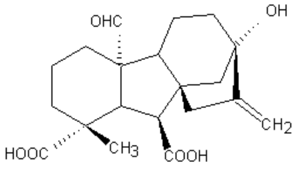 $GA_{19}$ | Mature seed of radish[32] | Control of bolting[32] |
|  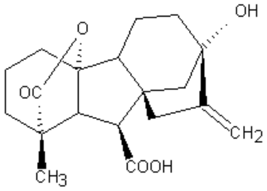 $GA_{20}$ | Mature seed of radish[32] | Control of bolting[32] |
|  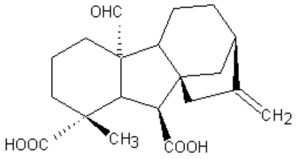 $GA_{24}$ | Mature seed of radish[32] | Control of bolting[32] |
|  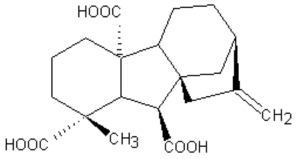 $GA_{25}$ | Mature seed of radish[32] | Control of bolting[32] |
|  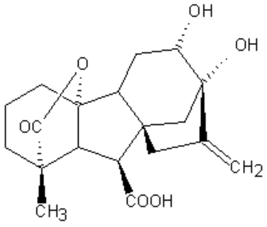 $GA_{77}$ | Mature seed of radish[32] | Control of bolting[32] |

**TABLE 5**
**Organic Acid Isolated from Radish**

| Structure | Source | Activities |
|---|---|---|
|  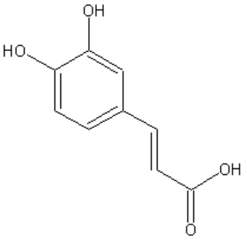 Caffeic acid | Leaves[18] | Antioxidative, anti-HIV, antineoplastic, choleretic, hepatotropic, and antifungal properties[41] |
|  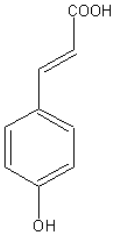 p-Coumaric acid | Leaves[18] | |
|  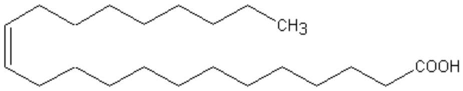 Erucic acid | Leaves and roots[41] | |
|  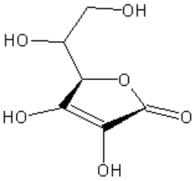 Erythorbic acid | Leaves and roots[41] | |
|  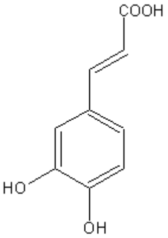 Ferulic acid | Leaves[41] | Antifungical[41] |
|  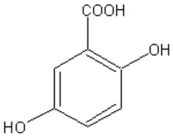 Gentisic acid | Leaves and roots[41] | Aanalgesic, antiarthritic, antirheumatic, and cytostatic[76] |
|  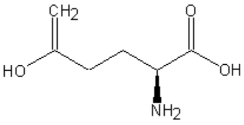 Glutamic acid | Pickled daikon[18] | |

**TABLE 5 (CONTINUED)**
**Organic Acid Isolated from Radish**

| Structure | Source | Activities |
|---|---|---|
|  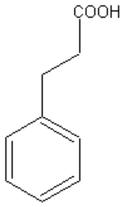 Hydrocinnamic acid | Leaves and roots[41] | Antimicrobial[76] |
|  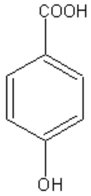 p-Hydroxybenzoic | Leaves and roots[18] | Antimicrobial[18] |
|  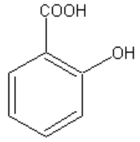 Salicylic acid | Leaves and roots[41] | Antifungical[76] |
|  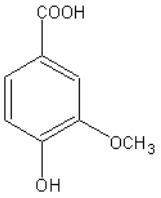 Vanillic acid | Leaves and roots[41] | Antimicrobial[41] |
| Linoleic, linolenic, malic, malonic, oleic, oxalic, and palmitic acid | Leaves and roots[39] | |

**TABLE 6**
**Phenolic Compounds Isolated from Radish**

| Structure | Source | Activities |
|---|---|---|
|  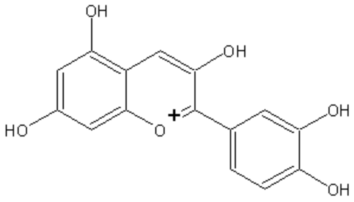 Cyanidin | Leaves and roots[42] | Antiulcer[76] |

**TABLE 6 (CONTINUED)**
**Phenolic Compounds Isolated from Radish**

| Structure | Source | Activities |
|---|---|---|
|   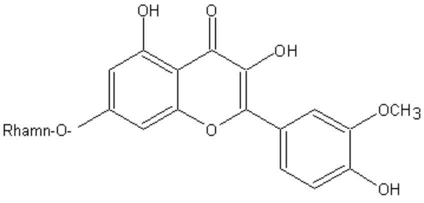 Isorhamnetin-7-O-rhamnoside | Roots, leaves, and green cotyledons[43] | |
|   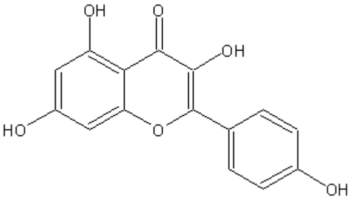 Kaempferol | Roots and leaves[44] | Antiinflammatory, diuretic, and antioxidant[76] |
|   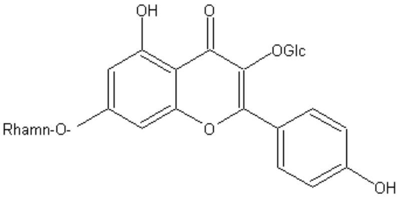 Kaempferol-3-glucoside-7-rhamnoside | Upper epidermis of the cotyledons[49] | Antiinflammatory[76] |
|   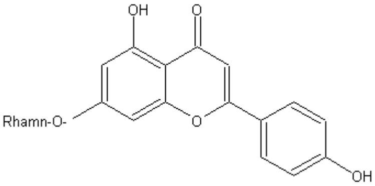 Kaempferol-7-O-rhamnoside | Roots[49] | Antiinflammatory[49] |
|   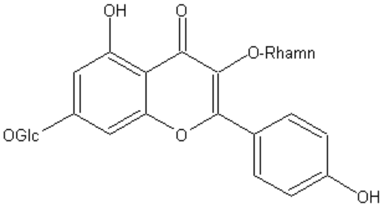 Kaempferol-7-glucoside-3-rhamnoside | Roots[48] | Antiinflammatory[76] |
|   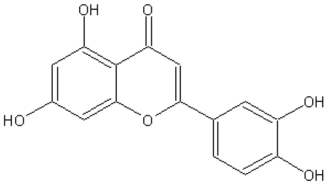 Luteolin | Roots[50] | Antiinflammatory, antitussive, antispasmodic, antitumourigenic, and anti-HIV activities[76] |

**TABLE 6 (CONTINUED)**
**Phenolic Compounds Isolated from Radish**

| Structure | Source | Activities |
|---|---|---|
| 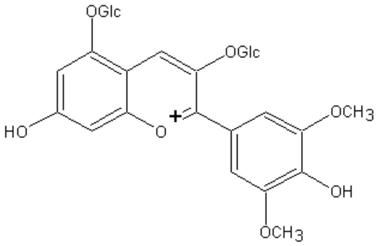 <br>Malvidin-3,5-diglucoside | Callus[51] | |
| 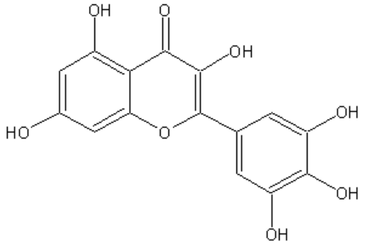 <br>Myricetin | Roots[50] | Anti-HIV activity[76] |
| 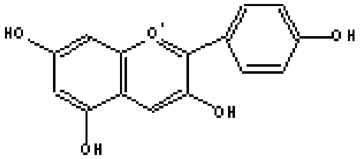 <br>Pelargonidin | Roots and fruits[47] | |
| 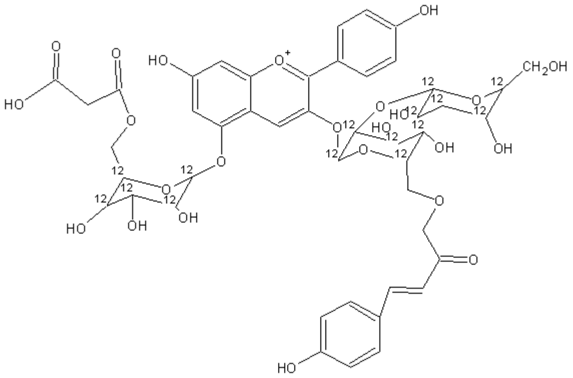 <br>Pelargonidin-3-O-[2-O-(β-glucopyranosyl)-6-O-(*trans*-p-coumaryl)-β-glucopyranoside]-5-O-(6-O-malonyl-β-glucopyranoside) | Roots[45] | Antioxidant[76] |

**TABLE 6 (CONTINUED)**
**Phenolic Compounds Isolated from Radish**

| Structure | Source | Activities |
|---|---|---|
|  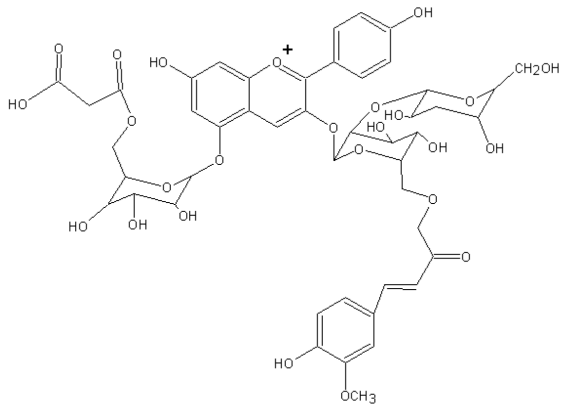 Pelargonidin-3-O-[2-O-(β-glucopyranosyl)-6-O-(*trans*-feruloyl)-β-glucopyranoside]-5-O-(6-O-malonyl-β-glucopyranoside) | Roots[45] | Antioxidant[76] |
|  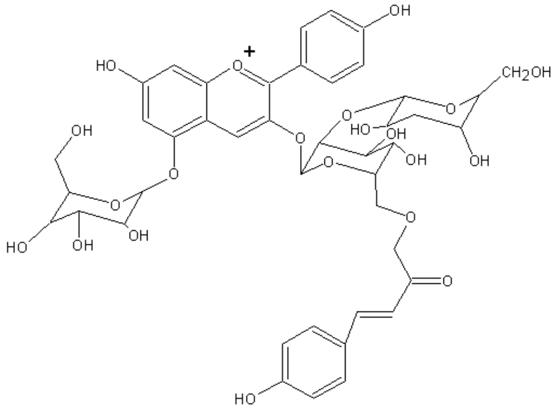 Pelargonidin 3-O-[2-O-β-glucopyranosyl)-6-O-(*trans*-p-coumaroyl)-β-D-glucopyranoside]-5-O-(β-glucopyranoside) | Roots[45] | Antioxidant[76] |
|  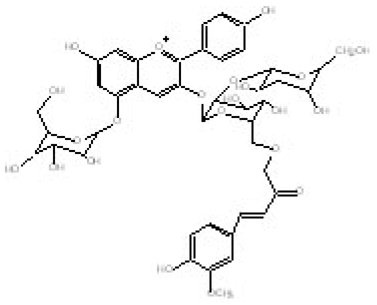 Pelargonidin 3-O-[2-O-β-glucopyranoside)-6-O-(*trans*-p-feruloyl)-β-glucopyranoside]-5-O-(β-glucopyranoside). | Roots[45] | Antioxidant activity[76] |

**TABLE 6 (CONTINUED)**
**Phenolic Compounds Isolated from Radish**

| Structure | Source | Activities |
|---|---|---|
|  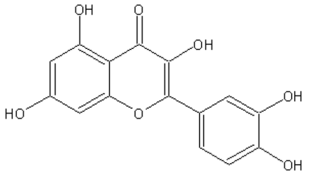 Quercetin | Roots and corollas[50] | Anticarcinogenic, antitumour promotor, and anti-HIV activities[50] |
|  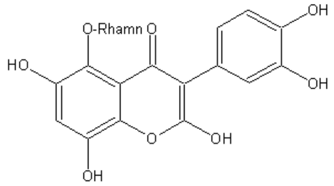 Quercetin-7-O-rhamnoside | Roots[49] | |
|  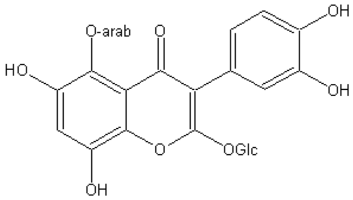 Quercetin-7-O-arabinoside-3-glucoside | Roots and corollas[49] | |
|  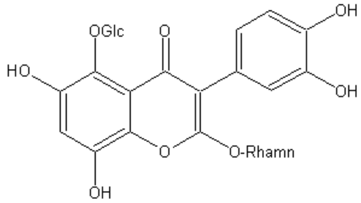 Quercetin-7-O-glucoside-3-rhamnoside | Bud and flowers[49] | |
|  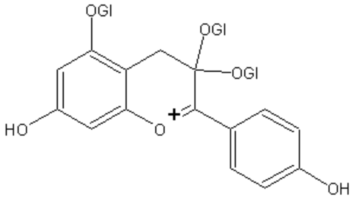 Raphanusin | Roots[47] | |

**TABLE 7**
**Polysaccharides Isolated from Radish**

| Structure | Source | Activities |
|---|---|---|
| Lipopolysaccharides (LPS) | Roots[54] | Antidiabetic, antiherpes, analgesic, and having a macrophage activation[76] |
| Pectic substances | Leaves[53] | |
| Proteoglycan | Seeds[55] | |
| Rhamnose, glucose, and xylose | Leaves[53] | |

**TABLE 8**
**Proteins Isolated from Radish**

| Structure | Source | Activities |
|---|---|---|
| Arabinogalactan-proteins (AGPs) | Mature leaves[10] | Reacted with eel anti-H serum[10] |
| L- Arabino-D-galactan<br>Arabino-3,6-galactan | Mature leaves[11] | Reacted with eel anti-H serum[11] |
| Chitinase RRC-A<br>Chitinase RRC-B | Roots[9] | |
| Cysteine-rich peptides Rs-AFP1 and Rs-AFP2 | Roots[17] | Antimicrobial[17] |
| Ferredoxin isoproteins | Leaves and roots[14] | |
| S-Glycoproteins | Mature leaves[13] | |
| Myrosinase | Daikon[8] | |
| Polypeptides RCA1, RCA2, and RCA3 | Mature leaves[15] | Calmodulin antagonist[15] |
| Proteins AGPs | Mature leaves[10] | Serological H-like activity[10] |
| Proteins RAP-1 and RAP-2 | Seeds[63] | Antimicrobial[63] |
| Protein AFP1 | Seeds[17] | Antifungical[17] |

**TABLE 9**
**Sulfur Isolated from Radish**

| Structure | Source | Activities |
|---|---|---|
| $H_2C{=}CHCH_2NCS$<br>Allyl-isothiocyanate | Leaves[59] | Produce allergic contact and dermatitis[59] |
| 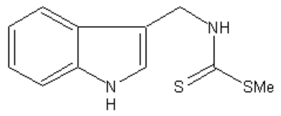 <br>Brassinin | Phytoalexin in daikon[64] | Antifungical[76] |

**TABLE 9 (CONTINUED)**
**Sulfur Isolated from Radish**

| Structure | Source | Activities |
|---|---|---|
| 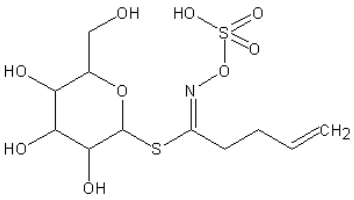 <br>Gluconapin | Oil radish seeds[38] | |
| Glucosinolate | Seeds[34] | |
| 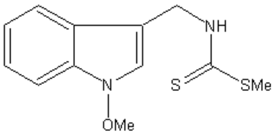 <br>Methoxybrassinin | Phytoalexin in daikon[64] | Antifungical[76] |
| 6-Methyl-sulfinylhexyl-isothiocianate | Leaves and roots[69] | Inhibitor of human platelet aggregation[69] |
| Myrosinase | Daikon[33] | |
| 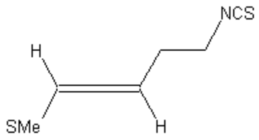 <br>*trans*-4-Methylthio-3-butenyl-isothiocyanate | Roots[72] | Pungent[72] |
| 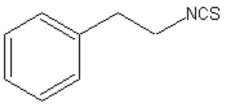 <br>Phenethylisothiocyanate | Roots[56] | |
| 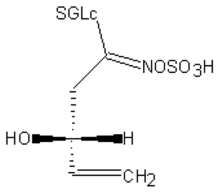 <br>Progoitrin | Oil radish seeds[38] | |
| 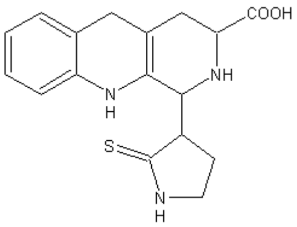 <br>1-(2´-Pyrrolidinethion-3´-yl)-1,2,3,4-tetrahydro-β-carboline-3-carboxilic acid | Root[35] | |

**TABLE 9 (CONTINUED)**
**Sulfur Isolated from Radish**

| Structure | Source | Activities |
|---|---|---|
|   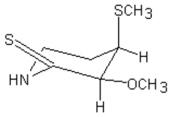 *cis*-Raphanusanin   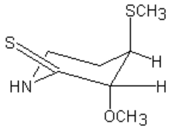 *trans*-Raphanusanin | Oil radish seeds[34] | |
| $CH_2$=$CHCH_2CH_2C(SGLc)$=$NOSO_3H$ Sinigrin | Leaves[33] | Produce allergic contact and dermatitis[33] |
|   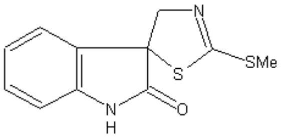 Spirobrassinin | Phytoalexin in daikon[64] | Antifungical[76] |
|   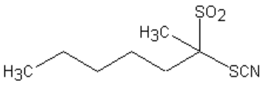 (S)-sulfonium form of S-adenosylmethionine | Immature leaves[56] | |
|   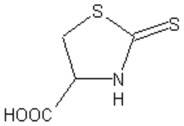 2-thioxothiazolidine-4-carboxilic acid | Roots[61] | Antimicrobial, inhibited the growth of hypocotyls[61] |
|   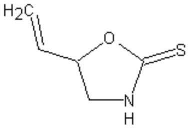 5-Vinyl-2-oxazolidinethione | Immature leaves[61] | |
| Other sulfurs: Isothiocyanate Dimethyl disulfide Methyl methanethiolsulfinate 1-Methylthio-3-pentanone | Oil radish seeds[38] | Biocidal and fungicidal activity[38] |

**TABLE 10**
**Other Compounds Isolated from Radish**

| Structure | Source | Activities |
|---|---|---|
| Carotenoids | Leaves[57] | Antioxidant[76] |
| Fatty acids | Seeds[8] | |
|  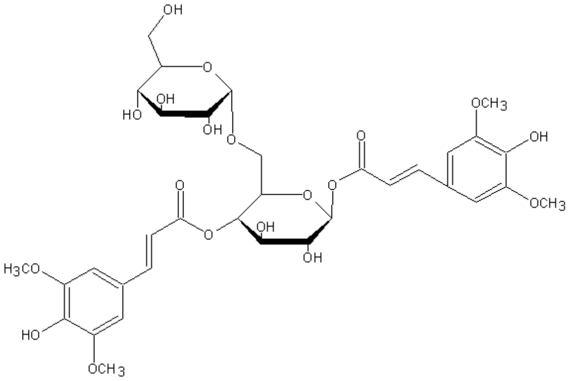 Raphanusol A | Leaves[68] | Growth inhibitor[68] |
|  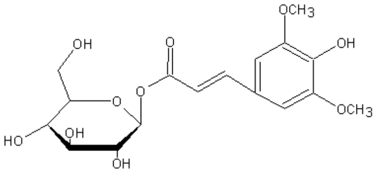 Raphanusol B | Leaves[68] | Growth inhibitor[68] |
| Pentyl<br>Hexyl<br>4-Methylpentyl | Oil radish seeds[37] | |
